# Health related quality of life in colorectal cancer patients: state of the art

**DOI:** 10.1186/1471-2482-13-S2-S15

**Published:** 2013-10-08

**Authors:** Stefano Marventano, Maria Joao Forjaz, Giuseppe Grosso, Antonio Mistretta, Gabriele Giorgianni, Alessio Platania, Santi Gangi, Francesco Basile, Antonio Biondi

**Affiliations:** 1 Department "G. F. Ingrassia" Section of Hygiene and Public Health, University of Catania, Catania, Italy; 2 National School of Public Health, Carlos III Institute of Health, Madrid, Spain; REDISSEC, Spain; 3 Department of Drug Sciences, Section of Biochemistry, University of Catania, Catania, Italy; 4 Department of General Surgery, Section of General Surgery and Oncology, University Medical School of Catania, Italy

## Abstract

**Background:**

Colorectal cancer (CRC) is the third most commonly diagnosed cancer in males and the second in females with a progressive increase in prevalence in industrialized countries. The loss of health due to the cancer and/or the consequence of the treatment may result in psychophysical, functional and social impairment; all of these affect health-related quality of life (QoL).

**Description:**

The most frequently CRC-specific QoL questionnaires is the FACT-C. QoL is not only important for the well-being of cancer patient but it also influences survival and response to therapy. Many studies investigated various determinants involved in the assessment of QoL in CRC, suggesting that symptoms, surgical procedures and the number of comorbidity significantly affected QoL.

**Conclusion:**

Despite that CRC patients have a relatively good QoL compared with the general population, a wide range of intervention could be undertaken to improve their QoL. The finding of this review may be useful for cancer clinicians in taking therapy and surveillance-related decisions. However, future research should be directed to large-scale prospective studies using well validated QoL instruments to facilitate comparison of results.

## Background

Colorectal cancer (CRC) is the third most commonly diagnosed cancer in males and the second in females, with over 1.2 million new cancer cases [1]. In the past two decades incidence rates for CRC have remained largely unchanged, instead mortality rates have fallen due to improvements in early detection and cancer treatment [2,3]. Survival at 5 years is 56% in Europe and 66% in the United States of America [4]. Moreover, Baade et al. concluded that survival expectations increase the longer they survive, reaching 93.2 % at 5 years after diagnosis [5]. This leads to a rising prevalence of patient living with CRC with an estimated worldwide prevalence of more than 3 million persons within 5 years of diagnosis in 2008 [6]. The rise of patients living with the consequence of CRC and its treatment has increased greatly the interest of their impact on health-related quality of life (QoL) [7]. The loss of health due to the cancer and/or the consequence of the treatment may result in psychophysical or functional impairment or disruption of social and family interactions, all of these affect QoL [8]. Several studies assess prospectively the impact of CRC in the patient's QoL, both in short-term [9,10] and long-term periods [11].

In the present article, we review the studies in colorectal cancer that have incorporated previously validated instruments.

## Quality of life: definition and assessment

QoL is a multimensional, dynamic, subjective and centered on patient construct, comprising physical, functional, emotional, and social/family well-being [12]. Therefore, QoL is an important outcome for evaluating the full impact of the disease on the individuals, their family and their community [13].

Quality of life, being a subjective, patient-rated concept, is difficult to quantify. To assess QoL the use of patient-reported questionnaires has become a standard practice. However the lack of a "gold standard" instrument is reflected in the wide range of available instruments, generic or disease-specific. The most used questionnaires are presented in table 1. Short Form 36 (SF-36) [14], its short version (SF-12) [15] and the EQ-5D [16] are the some of the most frequent generic questionnaire used to assess QoL. The use of generic QoL instruments allows comparison with the general population, or with people with no cancer. Instead, some cancer-specific questionnaires are the Functional Assessment of Cancer Therapy-General (FACT-G) [17] and the European Organization for the Research and Treatment of Cancer QLQ-C30 (EORTC QLQ-C30) [18]. The FACT-C [17] is the most used CRC specific questionnaire, although the EORTC also has a CRC-specific module, the QLQ-CR29 [19]. The FACT-C, used together with the FACT-G, has been extensively validated in English [20], Spanish [21] Korean [22], French [23] and Chinese patients [24]. It is formed by 37 items, grouped in five dimensions with a time frame of seven days. The first four dimensions are subscales of the FACT-G [20] (physical, social/family, emotional and functional well-being) and the last is an additional one focused on CRC. All the items are based on a five-point Likert scale except for the one investigating the presence of stoma (yes/no). This questionnaire was principally designed for self-administration, but it can also be administered by interviewers [25]. Both total and single dimensions scores could be calculated to assess QoL in CRC patient.

**Table 1:** List of QoL tools frequently used in oncology

Name of instruments	Type
SF-36 [14] and SF-12 [15]	Generic
EQ-5D [16]	Generic
FACT-G [17]	Cancer specific
EORTC QLQ-C30 [18]	Cancer specific
QoL-CS [84]	Cancer specific
EORTC QLQ-CR29 [19]	CRC specific
mCOH-QOL and abridged version for non-stoma patients [85]	CRC specific
FACT-C [17]	CRC specific

EORTC= European Organization for the Research and Treatment of Cancer; FACT= Functional Assessment of Cancer Therapy; mCOH= Modified City of Hope Quality of Life-Ostomy

## Determinant of quality of life

QoL in colorectal cancer patients is associated with a several number of factors. To simplify the discussion, we divided these factors into five broad categories: socio-demographic characteristics; health-related factors; cancer-related and surgical procedures factors; lifestyle factors; and other factors.

### Socio-demographic characteristics

Gender has not been reported as significant determinant in patients' QoL [26]. However, this is not true for specific problems like sexual functioning in man [27] or physical problems and pains in woman [28].

Results on age and CRC QoL are controversial. Forsberg et al. reported that age did not play a significant role in patients' QoL [29]. Nevertheless, in some studies QoL increase with age [30,31], whereas others reported a lower QoL with increasing age [32,33]. This controversy is present for both physical and psychological aspect of QoL [34].

Education level is not a determinant for QoL, because its role is subordinated to income [35]. With regards to income, there is evidence that low income correlates with worse physical, social and emotional well-being dimensions of QoL[11,31,35]. Also, home ownership was an independent predictor of QoL score [36].

The presence of a wide social network is positively related to patient's QoL [32]. Patients living alone reported a lower perceived well-being than those who live with family [29], but marital status was not associated with a higher QoL [11].

### Health-related factors

Patient with CRC reported significantly more comorbidity conditions and poorer physical and mental QoL compared with patients without cancer and a worse effect was found in patient with two or more comorbidities or those who had recent diagnosis [37]. Some specific diseases, such as heart disease [38], anxiety/depression [36] or urinary disorder [30] had a significant role on overall QoL. In particular, the higher prevalence of depression, compared to the general population of similar age [11] could be partially explained by the worries of a recurrence or of a second cancer, even after 5 or more years after diagnosis of cancer [39].

Concerning the association between body mass index and QoL, healthy-weight and overweight cancer survivors reported better scores in physical functioning, general health and vitality than obese cancer survivors [40].

### Cancer-related and surgical procedures factors

The stage and site of colorectal cancer at diagnosis are important in determining QoL, as they determine symptoms, treatment modalities and therapy duration [41,42]. Patient with stage I experienced a progressive positive trend in QoL; on the contrary patient with stage IV experienced a negative one. Instead, an initial QoL decrease, followed by better QoL scores, was experienced by those with stage II and stage III [43]. A possible explanation could be the perception (or re-conceptualization) of QoL after CRC diagnosis [44]. Nevertheless other studies reported no association between tumor stage and QoL [11,30].

Surgical procedures can affect various aspects of QoL due to physical and psychological consequences. Patients undergoing surgical procedures experimented a rapid QoL decline after surgery with a gradually restore about 3 month after [45]. Moreover, older patients are slower to restore their QoL [46].

Lower QoL both in laparoscopic and open surgery was found in older patient compared to the younger one [46], but only short-term QoL differences were found between open and laparoscopic surgery [47]. A possible reason may be the different occurrence of complications between the two surgical techniques [48]. Furthermore, baseline QOL was an important predictor of postoperative overall QOL and all QOL subdomains with a higher risk for difficult postoperative courses and auxiliary services associated with poor baseline QoL [49].

An important consequence of colorectal surgery is stoma. The presence of stoma influenced negatively the QOL if compared with patients undergoing a sphincter-saving resection [50], but not all authors found a significant difference [9,51]. The most important aspect influenced by the presence of stoma was the social component of QoL as assessed by a recent systematic review conducted on 10 studies [52].

The physical and psychological disorders resulting from stoma vary by gender. In female patients, a worse psychological [28] and physical [32] QoL score was reported. On the contrary, a reduction in mental health [28] and sexual functioning [53] was found in man. These and other problems related to stoma, such as worse fatigue, dyspnea, loss of appetite and changing in body image perception, gradually reduce a person's confidence and his social relations [54]. However, the impact of stoma could be influenced negatively by low income and problems in paying for stoma supplies [55], and positively by receiving therapy support with stoma-education programs and counseling [56,57].

Symptoms induced by cancer or its treatment are also very important. Many prospective studies investigated the role of bowel symptoms such as diarrhea, fecal control and constipation [58,59], but also fatigue and loss of appetite are very common and affected significantly QoL in CRC [36].

### Other factors

Some lifestyle factors such as physical activities, diet, alcohol intake and smoke were associated to QoL. A moderate or intense physical activity is correlated to a higher physical QoL due to lower levels of fatigue and distress [60]. A quality diet (rich in fruit and vegetables, and low in fat) and the administration of probiotics reduced bowel dysfunction, which can markedly decrease QoL [61-64]. Smoking was associated with a lower QoL [65], and controversy to alcohol intake [30]. Multiple behaviors changes have a better cumulative effect on QoL than single lifestyle modifications [63,66].

## Intervention to improve QoL in CRC

In order to improve QoL in CRC patients it is important to, first identify the patient with a higher risk to have a low QoL and then intervene to the modifiable factors.

Fixed factors such as age or sex have only a marginal role in QoL and others are potentially modifiable. Therefore, a wide range of interventions have been developed to improve QoL in CRC survivors. We can improve QoL by reducing psychological morbidity and facilitating crisis adaptation with educational programs, self-help groups, psychosocial interventions, cognitive behavioral therapy, coping, and certainly drugs. For example, a randomized trial on 200 with cancer found an improvement in depression and anxiety by physical exercise, intervention group therapy and antidepressants [67]. A moderate physical activities should be suggested, when possible, to reduce some symptoms such as fatigue, pain and insomnia [64]. Bowel symptoms could be reduced with modification of diet and the use of probiotics [62-64].

Psychosocial interventions can be classified in educational programs and psychotherapeutic interventions. Educational problems improve the cancer-related knowledge, its treatment and the emotional reactions to it. Psychotherapeutic interventions, individual or in groups, covers a wide range of approaches including assistance in expressing emotions, increasing patients' sense of coherence, enhancing personal resources, improving communication, gaining control and improving coping skills. These interventions help to decrease somatic symptoms and psychosocial sequelae of CRC and its treatment, and improving QoL as showed on a prospective study [68].

This is also true for surgery consequences. Specific training, like anal training after rectal resections by reducing stool frequency, improves both general and specific QoL [69]. A cross-sectional study and a recent systematic review found that education of patient living with stoma helped to deal with sexual difficulties, dietary and physical activity restriction and in general with their lifestyle [57,70]. Furthermore, in a prospective longitudinal study was showed that groups' activities by sharing personal experiences helps to reduce the isolation and the feelings of loneliness leading by stoma [71].

## Relationship to survival

QoL is also known to be an independent predictor of survival and response to therapy in cancer patients [72,73]. Broun et al. found that a 10-point increase in baseline global QoL scores (using EORTC QLQ-C30) was associated with a 7% decreased risk of death [74]. This result was also showed for other types of cancer [75,76]. Some authors proposed a theory according to which QoL could have a direct influence on tumor behavior and survival [77,78], others suggested that QoL had a direct influence on therapy adherence and consequently on survival [79]. Moreover, a recent 18-month trial suggested that baseline QoL influenced CRC patient's survival [49].

## Discussion and conclusion

Various determinants of QoL in CRC had been investigated and the results mainly shown that physical problems linked to symptoms and surgical procedures, such as bowel problems and stoma are the most common. On the socio-demographic characteristics, only the socioeconomic status seems to have a well determined role, probably due to the better access to medical care. The presence of a higher comorbidity number was the most important health-related factor, but it must be considered that some of them could be a consequence of CRC. Moreover, a significant higher prevalence of distress, depression and anxiety were reported in CRC patients than the general population.

Findings on CRC stage by determining symptoms, treatment procedure and consequently overall survival showed the importance of disease stage on QoL. Its role is essential both for the clinical aspect and the psychological consequences that affect QoL after diagnosis [43].

When comparing the effect of different surgical procedures on QoL, only short-term benefits were found for laparoscopy. This could be explained by less post-operative complications with laparoscopic procedure [2,46]. Similarly, higher QoL score for the younger patient was linked to the number and the severity of postoperative and late complications occurred after both open and laparoscopic surgery [46,48].

The importance of symptoms has been reported in many studies since they affect directly and indirectly QoL in CRC survival. In fact the presence of diarrhea, incontinence, fatigue and pain in addition to having direct effects on QoL influenced the daily activities and hobbies, and interfere with family and social life [36,59].

Despite specific physical and psychological problems, the overall QoL in CRC patient is good both in short [80] and long-term survivors [11,27]. Several theories can be called upon to explain these findings.

One could be the process of internal recalibration and the shift in personal values to new understanding, known as reframing/response shift. This changes the patient's internal standards leading to a different estimation of their QoL [81].

Other two constructs of positive consequences after cancer diagnosis/treatment describe the change following a so stressful experience, benefit finding (BF) and post-traumatic growth (PTG). These two concepts are slightly different, but sometimes have been used interchangeably [82]. BF is defined as an individual process in which the patient perceives that major positive changes have occurred as a result of challenging life events. In contrast, PTG refers to benefits associated with changes in appreciation of life, interpersonal relationship, and self-perception often manifested through personal strength, spiritual change and globally as life perspective. Moreover, these two concepts are temporally different. BF start immediately after diagnosis, instead PTG can be developed even years after the cancer diagnosis [83].

The limitations of a literature review of this nature are the lack of systematic research of articles and lack of a gold standard for measuring QoL. Very heterogeneous instruments and different statistical analyses were used, making difficult a comparison across studies. However, all studies used well-validated instruments. Other shortcomings of the exanimated studies which may introduce a bias when comparing QoL results are: data acquisition, low response rates, non-random drop-out, small sample size and the different correction for confounding factors. Furthermore role of chemotherapy and radiotherapy treatment was not investigated.

Despite some limitations, this review is useful for a better understanding of QoL and its determinants in CRC patients. Due to the burden of this disease and the higher survival rate, both for early diagnosis and new treatment, the QoL in survivors of CRC should be a priority for public health research. The knowledge about the determinants of QoL could help to identify survivors with special needs. Moreover these findings may be useful for cancer clinicians in taking therapy and surveillance-related decisions in order to enhance the QoL of people with CRC.

Finally, although patients have a good QoL compared with the general populations, a significant number of determinants are potentially modifiable variable. Future intervention studies are needed to improve certain aspects of quality of care to determine whether those changes lead to increased QoL. Moreover, research should be directed to large-scale prospective studies using well validated QoL instruments to facilitate the comparison of results.

## Competing interests

The authors declare that they have no competing interests.

## Authors' contributions

SM, MJF: conception and design, drafting the manuscript; GG, AM, GG, AP: drafting the manuscript; FB, SG, AB: critical revision, given final approval of the version to be published.

## Declarations

Funding for this article has come from University funds.

This article has been published as part of *BMC Surgery *Volume 13 Supplement 2, 2013: Proceedings from the 26th National Congress of the Italian Society of Geriatric Surgery. The full contents of the supplement are available online at http://www.biomedcentral.com/bmcsurg/supplements/13/S2

## References

[B1] Jemal A Bray F Center MM Ferlay J Ward E Forman D Global cancer statistics CA: a cancer journal for clinicians 2011 13 2 69 90 21296855 10.3322/caac.20107

[B2] Biondi A Grosso G Mistretta A Marventano S Toscano C Gruttadauria S Basile F Laparoscopic-assisted versus open surgery for colorectal cancer: short- and long-term outcomes comparison Journal of laparoendoscopic & advanced surgical techniques Part A 2013 13 1 1 7 23004676 10.1089/lap.2012.0276 PMC3698686

[B3] Biondi A Tropea A Basile F Clinical rescue evaluation in laparoscopic surgery for hepatic metastases by colorectal cancer Surgical laparoscopy, endoscopy & percutaneous techniques 2010 13 2 69 72 10.1097/SLE.0b013e3181d83f02 20393330

[B4] Verdecchia A Francisci S Brenner H Gatta G Micheli A Mangone L Kunkler I Recent cancer survival in Europe: a 2000-02 period analysis of EUROCARE-4 data The lancet oncology 2007 13 9 784 796 17714993 10.1016/S1470-2045(07)70246-2

[B5] Baade PD Youlden DR Chambers SK When do I know I am cured? Using conditional estimates to provide better information about cancer survival prospects Medical Journal of Australia 2011 13 73 21241220 10.5694/j.1326-5377.2011.tb04171.x

[B6] GLOBOCAN 2002: cancer incidence, mortality and prevalence worldwide http://www-dep.iarc.fr IARC CancerBase No. 5. version 2.0

[B7] Donaldson GW Moinpour CM Individual differences in quality-of-life treatment response Medical care 2002 13 6 Suppl III39 53 12064757 10.1097/00005650-200206001-00007

[B8] Frazzetto P Vacante M Malaguarnera M Vinci E Catalano F Cataudella E Drago F Malaguarnera G Basile F Biondi A Depression in older breast cancer survivors BMC surgery 2012 13 Suppl 1 S14 23173836 10.1186/1471-2482-12-S1-S14 PMC3499203

[B9] Smith-Gagen J Cress RD Drake CM Romano PS Yost KJ Ayanian JZ Quality-of-life and surgical treatments for rectal cancer--a longitudinal analysis using the California Cancer Registry Psycho-oncology 2010 13 8 870 878 19862692 10.1002/pon.1643 PMC2911491

[B10] Wilson TR Alexander DJ Kind P Measurement of healthrelated quality of life in the early follow-up of colon and rectal cancer Dis Colon Rectum 2006 13 1692 1702 17041750 10.1007/s10350-006-0709-9

[B11] Ramsey SD Berry K Moinpour C Giedzinska A Andersen MR Quality of life in long term survivors of colorectal cancer The American journal of gastroenterology 2002 13 5 1228 1234 12017152 10.1111/j.1572-0241.2002.05694.x

[B12] Cella DF Tulsky DS Quality of life in cancer: definition, purpose, and method of measurement Cancer investigation 1993 13 3 327 336 8485655 10.3109/07357909309024860

[B13] Sahay TB Gray RE Fitch M A qualitative study of patient perspectives on colorectal cancer Cancer practice 2000 13 1 38 44 10732538 10.1046/j.1523-5394.2000.81012.x

[B14] Ware JE Sherbourne CD The MOS 36-item short-form health survey (SF-36). I. Conceptual framework and item selection Medical care 1992 13 6 473 483 1593914

[B15] Ware J Kosinski M Keller SD A 12-Item Short-Form Health Survey: construction of scales and preliminary tests of reliability and validity Medical care 1996 13 3 220 233 8628042 10.1097/00005650-199603000-00003

[B16] Brooks R EuroQol: the current state of play Health Policy 1996 13 1 53 72 10158943 10.1016/0168-8510(96)00822-6

[B17] Cella DF Tulsky DS Gray G Sarafian B Linn E Bonomi A Silberman M Yellen SB Winicour P Brannon J The Functional Assessment of Cancer Therapy scale: development and validation of the general measure Journal of clinical oncology : official journal of the American Society of Clinical Oncology 1993 13 3 570 579 8445433 10.1200/JCO.1993.11.3.570

[B18] Sprangers MA Cull A Bjordal K Groenvold M Aaronson NK The European Organization for Research and Treatment of Cancer. Approach to quality of life assessment: guidelines for developing questionnaire modules. EORTC Study Group on Quality of Life Quality of life research : an international journal of quality of life aspects of treatment, care and rehabilitation 1993 13 4 287 295 10.1007/BF00434800 8220363

[B19] Whistance RN Conroy T Chie W Costantini A Sezer O Koller M Johnson CD Pilkington SA Arraras J Ben-Josef E Clinical and psychometric validation of the EORTC QLQ-CR29 questionnaire module to assess health-related quality of life in patients with colorectal cancer Eur J Cancer 2009 13 17 3017 3026 19765978 10.1016/j.ejca.2009.08.014

[B20] Ward WL Hahn EA Mo F Hernandez L Tulsky DS Cella D Reliability and validity of the Functional Assessment of Cancer Therapy-Colorectal (FACT-C) quality of life instrument Quality of life research : an international journal of quality of life aspects of treatment, care and rehabilitation 1999 13 3 181 195 10.1023/a:1008821826499 10472150

[B21] Cella D Hernandez L Bonomi AE Corona M Vaquero M Shiomoto G Baez L Spanish language translation and initial validation of the functional assessment of cancer therapy quality-of-life instrument Medical care 1998 13 9 1407 1418 9749663 10.1097/00005650-199809000-00012

[B22] Yoo HJ Kim JC Eremenco S Han OS Quality of life in colorectal cancer patients with colectomy and the validation of the Functional Assessment of Cancer Therapy-Colorectal (FACT-C), Version 4 Journal of pain and symptom management 2005 13 1 24 32 16043004 10.1016/j.jpainsymman.2004.12.009

[B23] Rotonda C Conroy T Mercier M Bonnetain F Uwer L Miny J Montcuquet P Leonard I Adenis A Breysacher G Validation of the French version of the colorectal-specific quality-of-life questionnaires EORTC QLQ-CR38 and FACT-C Quality of life research : an international journal of quality of life aspects of treatment, care and rehabilitation 2008 13 3 437 445 10.1007/s11136-008-9322-9 18338238

[B24] Wong CK Lam CL Law WL Poon JT Chan P Kwong DL Tsang J Validity and reliability study on traditional Chinese FACT-C in Chinese patients with colorectal neoplasm Journal of evaluation in clinical practice 2012 13 6 1186 1195 21851512 10.1111/j.1365-2753.2011.01753.x

[B25] Webster K Cella D Yost K The Functional Assessment of Chronic Illness Therapy (FACIT) Measurement System: properties, applications, and interpretation Health Qual Life Outcomes 2003 13 79 14678568 10.1186/1477-7525-1-79 PMC317391

[B26] Dibble SL Padilla GV Dodd MJ Miaskowski C Gender differences in the dimensions of quality of life Oncology nursing forum 1998 13 3 577 583 9568612

[B27] Phipps E Braitman LE Stites S Leighton JC Quality of life and symptom attribution in long-term colon cancer survivors Journal of evaluation in clinical practice 2008 13 2 254 258 18284521 10.1111/j.1365-2753.2007.00842.x

[B28] Krouse RS Herrinton LJ Grant M Wendel CS Green SB Mohler MJ Baldwin CM McMullen CK Rawl SM Matayoshi E Health-related quality of life among long-term rectal cancer survivors with an ostomy: manifestations by sex Journal of clinical oncology : official journal of the American Society of Clinical Oncology 2009 13 28 4664 4670 19720920 10.1200/JCO.2008.20.9502 PMC2754912

[B29] Forsberg C Bjorvell H Cedermark B Well-being and its relation to coping ability in patients with colo-rectal and gastric cancer before and after surgery Scandinavian journal of caring sciences 1996 13 1 35 44 8715784 10.1111/j.1471-6712.1996.tb00308.x

[B30] Hamashima C Long-term quality of life of postoperative rectal cancer patients Journal of gastroenterology and hepatology 2002 13 5 571 576 12084031 10.1046/j.1440-1746.2002.02712.x

[B31] Klemm P Miller MA Fernsler J Demands of illness in people treated for colorectal cancer Oncology nursing forum 2000 13 4 633 639 10833692

[B32] Sapp AL Trentham-Dietz A Newcomb PA Hampton JM Moinpour CM Remington PL Social networks and quality of life among female long-term colorectal cancer survivors Cancer 2003 13 8 1749 1758 14534893 10.1002/cncr.11717

[B33] Trentham-Dietz A Remington PL Moinpour CM Hampton JM Sapp AL Newcomb PA Health-related quality of life in female long-term colorectal cancer survivors The oncologist 2003 13 4 342 349 12897331 10.1634/theoncologist.8-4-342

[B34] Vacante M D'Agata V Motta M Malaguarnera G Biondi A Basile F Malaguarnera M Gagliano C Drago F Salamone S Centenarians and supercentenarians: a black swan. Emerging social, medical and surgical problems BMC surgery 2012 13 Suppl 1 S36 23173707 10.1186/1471-2482-12-S1-S36 PMC3499197

[B35] Lundy JJ Coons SJ Wendel C Hornbrook MC Herrinton L Grant M Krouse RS Exploring household income as a predictor of psychological well-being among long-term colorectal cancer survivors Quality of life research : an international journal of quality of life aspects of treatment, care and rehabilitation 2009 13 2 157 161 10.1007/s11136-008-9432-4 PMC2637932 19132550

[B36] Gray NM Hall SJ Browne S Macleod U Mitchell E Lee AJ Johnston M Wyke S Samuel L Weller D Modifiable and fixed factors predicting quality of life in people with colorectal cancer British journal of cancer 2011 13 11 1697 1703 21559017 10.1038/bjc.2011.155 PMC3111166

[B37] Smith AW Reeve BB Bellizzi KM Harlan LC Klabunde CN Amsellem M Bierman AS Hays RD Cancer, comorbidities, and health-related quality of life of older adults Health care financing review 2008 13 4 41 56 18773613 PMC3142673

[B38] Ko CY Maggard M Livingston EH Evaluating health utility in patients with melanoma, breast cancer, colon cancer, and lung cancer: a nationwide, population-based assessment The Journal of surgical research 2003 13 1 1 5 13678691 10.1016/s0022-4804(03)00167-7

[B39] Deimling GT Bowman KF Sterns S Wagner LJ Kahana B Cancer-related health worries and psychological distress among older adult, long-term cancer survivors Psycho-oncology 2006 13 4 306 320 16041841 10.1002/pon.955

[B40] Blanchard CM Stein K Courneya KS Body mass index, physical activity, and health-related quality of life in cancer survivors Medicine and science in sports and exercise 2010 13 4 665 671 19952838 10.1249/MSS.0b013e3181bdc685

[B41] Paika V Almyroudi A Tomenson B Creed F Kampletsas EO Siafaka V Gkika S Mavreas V Pavlidis N Hyphantis T Personality variables are associated with colorectal cancer patients' quality of life independent of psychological distress and disease severity Psycho-oncology 2010 13 3 273 282 19353527 10.1002/pon.1563

[B42] Cardin F Andreotti A Zorzi M Terranova C Martella B Amato B Militello C Usefulness of a fast track list for anxious patients in a upper GI endoscopy BMC Surgery 2012 13 Suppl 1 S11 23173721 10.1186/1471-2482-12-S1-S11 PMC3499366

[B43] Ramsey SD Andersen MR Etzioni R Monipour CM Quality of life in survivors of colorectal carcinoma Cancer investigation 1999 13 6 1294 1303 10717609

[B44] Sprangers MA Schwartz CE Integrating response shift into health-related quality of life research: a theoretical model Soc Sci Med 1999 13 11 1507 1515 10400253 10.1016/s0277-9536(99)00045-3

[B45] Schmidt CE Bestmann B Kuchler T Longo WE Kremer B Ten-year historic cohort of quality of life and sexuality in patients with rectal cancer Dis Colon Rectum 2005 13 3 483 492 15747079 10.1007/s10350-004-0822-6

[B46] Scarpa M Di Cristofaro L Cortinovis M Pinto E Massa M Alfieri R Cagol M Saadeh L Costa A Castoro C Minimally invasive surgery for colorectal cancer: quality of life and satisfaction with care in elderly patients Surgical endoscopy 2013 10.1007/s00464-013-2854-2 23468328

[B47] Weeks JC Nelson H Gelber S Sargent D Schroeder G Short-term quality-of-life outcomes following laparoscopic-assisted colectomy vs open colectomy for colon cancer: a randomized trial JAMA : the journal of the American Medical Association 2002 13 3 321 328 11790211 10.1001/jama.287.3.321

[B48] Grosso G Biondi A Marventano S Mistretta A Calabrese G Basile F Major postoperative complications and survival for colon cancer elderly patients BMC surgery 2012 13 Suppl 1 S20 23173563 10.1186/1471-2482-12-S1-S20 PMC3499273

[B49] Stucky CC Pockaj BA Novotny PJ Sloan JA Sargent DJ O'Connell MJ Beart RW Skibber JM Nelson H Weeks JC Long-term follow-up and individual item analysis of quality of life assessments related to laparoscopic-assisted colectomy in the COST trial 93-46-53 (INT 0146) Annals of surgical oncology 2011 13 9 2422 2431 21452066 10.1245/s10434-011-1650-2 PMC3947623

[B50] Rispoli C Rocco N Iannone L Amato B Developing guidelines in geriatric surgery:role of the grade system BMC Geriatrics 2009 13 SUPPL.1 A99

[B51] De Campos-Lobato LF Alves Ferreira PC Lavery IC Kira RP Abdominoperineal resection does not decrease quality of life in patients with low rectal cancer Clinics 2001 13 6 1035 1040 10.1590/S1807-59322011000600019 PMC3129949 21808871

[B52] Jansen L Koch L Brenner H Arndt V Quality of life among long-term (>/ = 5 years) colorectal cancer survivors--systematic review Eur J Cancer 2010 13 16 2879 2888 20605090 10.1016/j.ejca.2010.06.010

[B53] Guren MG Eriksen MT Wiig JN Carlsen E Nesbakken A Sigurdsson HK Wibe A Tveit KM Quality of life and functional outcome following anterior or abdominoperineal resection for rectal cancer European journal of surgical oncology : the journal of the European Society of Surgical Oncology and the British Association of Surgical Oncology 2005 13 7 735 742 16180267 10.1016/j.ejso.2005.05.004

[B54] Dabirian A Yaghmaei F Rassouli M Tafreshi MZ Quality of life in ostomy patients: a qualitative study Patient preference and adherence 2010 13 1 5 21311696 10.2147/PPA.S14508 PMC3034300

[B55] Coons SJ Chongpison Y Wendel CS Grant M Krouse RS Overall quality of life and difficulty paying for ostomy supplies in the Veterans Affairs ostomy health-related quality of life study: an exploratory analysis Medical care 2007 13 9 891 895 17712260 10.1097/MLR.0b013e318074ce9b

[B56] Celasin H Karakoyun R Yilmaz S Elhan AH Erkek B Kuzu MA Quality of life measures in Islamic rectal carcinoma patients receiving counselling Colorectal disease : the official journal of the Association of Coloproctology of Great Britain and Ireland 2011 13 7 e170 175 21651692 10.1111/j.1463-1318.2011.02649.x

[B57] Danielsen AK Burcharth J Rosenberg J Patient education has a positive effect in patients with a stoma: a sytematic review Colorectal disease : the official journal of the Association of Coloproctology of Great Britain and Ireland 2013 10.1111/codi.12197 23470040

[B58] Steginga SK Lynch BM Hawkes A Dunn J Aitken J Antecedents of domain-specific quality of life after colorectal cancer Psycho-oncology 2009 13 2 216 220 18618899 10.1002/pon.1388

[B59] Pan LH Tsai YF Quality of life in colorectal cancer patients with diarrhoea after surgery: a longitudinal study Journal of clinical nursing 2012 13 156 2357 2366 22632065 10.1111/j.1365-2702.2011.04034.x

[B60] Buffart LM Thong MS Schep G Chinapaw MJ Brug J van de Poll-Franse LV Self-reported physical activity: its correlates and relationship with health-related quality of life in a large cohort of colorectal cancer survivors PloS one 2012 13 5 e36164 22567135 10.1371/journal.pone.0036164 PMC3342261

[B61] Uccello M Malaguarnera G Basile F D'Agata V Malaguarnera M Bertino G Vacante M Drago F Biondi A Potential role of probiotics on colorectal cancer prevention BMC surgery 2012 13 Suppl 1 S35 23173670 10.1186/1471-2482-12-S1-S35 PMC3499195

[B62] Ohigashi S Hoshino Y Ohde S Onodera H Functional outcome, quality of life, and efficacy of probiotics in postoperative patients with colorectal cancer Surgery today 2011 13 9 1200 1206 21874415 10.1007/s00595-010-4450-6

[B63] Amato B Rispoli C Iannone L Testa S Compagna R Rocco N Surgical margins of resection for breast cancer: Current evidence Minerva Chirurgica 2012 13 5 445 452 23232484

[B64] Grimmett C Bridgewater J Steptoe A Wardle J Lifestyle and quality of life in colorectal cancer survivors Quality of life research : an international journal of quality of life aspects of treatment, care and rehabilitation 2011 13 8 1237 1245 10.1007/s11136-011-9855-1 21286822

[B65] Blanchard CM Stein KD Baker F Dent MF Denniston MM Courneya KS Nehl E Association between current lifestyle behaviors and health-related quality of life in breast, colorectal, and prostate cancer survivors Psychology & Health 2004 13 1 1 13

[B66] Blanchard CM Courneya KS Stein K Cancer survivors' adherence to lifestyle behavior recommendations and associations with health-related quality of life: results from the American Cancer Society's SCS-II Journal of clinical oncology : official journal of the American Society of Clinical Oncology 2008 13 13 2198 2204 18445845 10.1200/JCO.2007.14.6217

[B67] Strong V Waters R Hibberd C Murray G Wall L Walker J McHugh G Walker A Sharpe M Management of depression for people with cancer (SMaRT oncology 1): a randomised trial Lancet 2008 13 9632 40 48 18603157 10.1016/S0140-6736(08)60991-5

[B68] Pugliese P Perrone M Nisi E Garufi C Giannarelli D Bottomley A Terzoli E An integrated psychological strategy for advanced colorectal cancer patients Health Qual Life Outcomes 2006 13 9 16460566 10.1186/1477-7525-4-9 PMC1409769

[B69] Laforest A Bretagnol F Mouazan AS Maggiori L Ferron M Panis Y Functional disorders after rectal cancer resection: does a rehabilitation programme improve anal continence and quality of life? Colorectal disease : the official journal of the Association of Coloproctology of Great Britain and Ireland 2012 13 10 1231 1237 22268662 10.1111/j.1463-1318.2012.02956.x

[B70] Anaraki F Vafaie M Behboo R Maghsoodi N Esmaeilpour S Safaee A Quality of life outcomes in patients living with stoma Indian journal of palliative care 2012 13 3 176 180 23439841 10.4103/0973-1075.105687 PMC3573471

[B71] Altuntas YE Kement M Gezen C Eker HH Aydin H Sahin F Okkabaz N Oncel M The role of group education on quality of life in patients with a stoma European journal of cancer care 2012 13 6 776 781 22672332 10.1111/j.1365-2354.2012.01360.x

[B72] Roychowdhury DF Hayden A Liepa AM Health-related quality-of-life parameters as independent prognostic factors in advanced or metastatic bladder cancer Journal of clinical oncology : official journal of the American Society of Clinical Oncology 2003 13 4 673 678 12586805 10.1200/JCO.2003.04.166

[B73] Maisey NR Norman A Watson M Allen MJ Hill ME Cunningham D Baseline quality of life predicts survival in patients with advanced colorectal cancer Eur J Cancer 2002 13 10 1351 1357 12091066 10.1016/s0959-8049(02)00098-9

[B74] Braun DP Gupta D Grutsch JF Staren ED Can changes in health related quality of life scores predict survival in stages III and IV colorectal cancer? Health and quality of life outcomes 2011 13 62 21812962 10.1186/1477-7525-9-62 PMC3162879

[B75] Meyer F Fortin A Gelinas M Nabid A Brochet F Tetu B Bairati I Health-related quality of life as a survival predictor for patients with localized head and neck cancer treated with radiation therapy Journal of clinical oncology : official journal of the American Society of Clinical Oncology 2009 13 18 2970 2976 19451440 10.1200/JCO.2008.20.0295

[B76] Luoma ML Hakamies-Blomqvist L Sjostrom J Pluzanska A Ottoson S Mouridsen H Bengtsson NO Bergh J Malmstrom P Valvere V Prognostic value of quality of life scores for time to progression (TTP) and overall survival time (OS) in advanced breast cancer Eur J Cancer 2003 13 10 1370 1376 12826039 10.1016/s0959-8049(02)00775-x

[B77] Coates AS Hurny C Peterson HF Bernhard J Castiglione-Gertsch M Gelber RD Goldhirsch A Quality-of-life scores predict outcome in metastatic but not early breast cancer. International Breast Cancer Study Group Journal of clinical oncology : official journal of the American Society of Clinical Oncology 2000 13 22 3768 3774 11078489 10.1200/JCO.2000.18.22.3768

[B78] Dancey J Zee B Osoba D Whitehead M Lu F Kaizer L Latreille J Pater JL Quality of life scores: an independent prognostic variable in a general population of cancer patients receiving chemotherapy. The National Cancer Institute of Canada Clinical Trials Group Quality of life research : an international journal of quality of life aspects of treatment, care and rehabilitation 1997 13 2 151 158 10.1023/a:1026442201191 9161115

[B79] Andreyev HJ Norman AR Oates J Cunningham D Why do patients with weight loss have a worse outcome when undergoing chemotherapy for gastrointestinal malignancies? Eur J Cancer 1998 13 4 503 509 9713300 10.1016/s0959-8049(97)10090-9

[B80] Rispoli C Rocco N Iannone L Amato B Developing guidelines in geriatric surgery:role of the grade system BMC Geriatrics 2009 13 SUPPL.1 A99

[B81] Bernhard J Lowy A Mathys N Herrmann R Hurny C Health related quality of life: a changing construct? Quality of life research : an international journal of quality of life aspects of treatment, care and rehabilitation 2004 13 7 1187 1197 10.1023/B:QURE.0000037485.59681.7d 15473497

[B82] Sumalla EC Ochoa C Blanco I Posttraumatic growth in cancer: reality or illusion? Clinical psychology review 2009 13 1 24 33 18996633 10.1016/j.cpr.2008.09.006

[B83] Calhoun LG Tedeschi RG Post-traumatic growth: future directions Posttraumatic Growth: Positive Changes in the Aftermath of Crisis 1998 Mahwaj, NJ: Lawrence Erlbaum Associates

[B84] Ferrell BR Dow KH Grant M Measurement of the quality of life in cancer survivors Quality of life research : an international journal of quality of life aspects of treatment, care and rehabilitation 1995 13 6 523 531 10.1007/BF00634747 8556012

[B85] Grant M Ferrell B Dean G Uman G Chu D Krouse R Revision and psychometric testing of the City of Hope Quality of Life-Ostomy Questionnaire Quality of life research : an international journal of quality of life aspects of treatment, care and rehabilitation 2004 13 8 1445 1457 10.1023/B:QURE.0000040784.65830.9f 15503840

